# Assessing adoption potential in a risky environment: The case of perennial pigeonpea

**DOI:** 10.1016/j.agsy.2019.01.001

**Published:** 2019-05

**Authors:** Philip Grabowski, Laura Schmitt Olabisi, Jelili Adebiyi, Kurt Waldman, Robert Richardson, Leonard Rusinamhodzi, Sieglinde Snapp

**Affiliations:** a Department of Plant, Soil and Microbial Sciences, Michigan State University, East Lansing, MI 48824, United States; b Department of Community Sustainability, Michigan State University, East Lansing, MI 48824, United States; c International Maize and Wheat Improvement Center (CIMMYT), P.O. Box 1041, Nairobi, Kenya

## Abstract

Perennial crops offer the opportunity to harvest from the same plant many times over several years while reducing labor and seed costs, reducing emissions and increasing biomass input into the soil. We use system dynamics modeling to combine data from field experiments, crop modeling and choice experiments to explore the potential for adoption and diffusion of a sustainable agriculture technology in a risky environment with high variability in annual rainfall: the perennial management of pigeonpea in maize-based systems of Malawi. Production estimates from a crop model for the annual intercrop system and data from field experiments on ratooning for the perennial system provided the information to create a stochastic production model. Data from choice experiments posed by a farmer survey conducted in three Malawi districts provide the information for parameters on farmers’ preferences for the attributes of the perennial system. The perennial pigeonpea technology appeared clearly superior in scenarios where average values for maize yield and pigeonpea biomass production were held constant. Adoption was fastest in scenarios where relatively dry growing seasons showcased the benefits of the perennial system, suggesting that perennial management may be appropriate in marginal locations. The potential for adoption was reduced greatly when stochasticity in yields and seasons combine with significant social pressure to conform. The mechanism for this is that low yields suppress adoption and increase disadoption due to the dynamics of trust in the technology. This finding is not unique to perennial pigeonpea, but suggests that a critical factor in explaining low adoption rates of any new agricultural technology is the stochasticity in a technology’s performance. Understanding how that stochasticity interacts with the social dynamics of learning skills and communicating trust is a critical feature for the successful deployment of sustainable agricultural technologies, and a novel finding of our study.

## Highlights

• Dynamic models of adoption can use choice experiments to parameterize preferences
• Perennial pigeonpea shows strong adoption potential, even on marginal land in Malawi
• Variable performance of a new technology can suppress adoption due to loss of trust

## 1 Introduction

Increasing soil fertility in sub-Saharan Africa is a major priority for funders, farmers, and agricultural development organizations (Vanlauwe et al., 2011). Improvements in soil health are recognized as a crucial prerequisite to reducing rural poverty, increasing farm output, and improving food security in Africa (Sanginga et al., 2009). Raising sub-Saharan Africa's historically low soil productivity will necessitate improved technologies that are suitable for, and are attractive to, the smallholders who cultivate most of the land in that region (McCalla, 1999).

One possibility is to improve degraded land through the perennial management of the leguminous shrub pigeonpea. Perennial grains in general have a number of potential benefits for the sustainability and resilience of farming systems, most of which apply to managing pigeonpea as a perennial. Once they are established, perennial crops have more and deeper roots than annuals, which enables the efficient uptake of soil nutrients and soil water (DeHaan et al., 2005; Glover et al., 2010; Pimentel et al., 2012; Kantar et al., 2016). This is especially advantageous during dry spells (Glover et al., 2012), which may become more frequent under climate change. Furthermore, the larger root systems of perennials have the potential to reduce soil erosion and contribute to increasing soil organic matter, which improves soil water holding capacity over the long term (Pimentel et al., 2012; Snapp, 2014). Ratooned pigeonpea plants are expected to have larger root systems, which can reduce the risk of erosion, especially early in the season. In addition to producing grains for food, perennial grains can also provide fodder for livestock (Snapp, 2014) and stover as fuel for cooking.

In recent years, there has been increased attention to technology uptake by smallholder farmers as an important aspect of mobilizing a ‘Green Revolution’ in Africa (Sanginga, 2010; Snapp et al., 2010). There is increasing recognition that technology performance is highly dependent on agro-ecological diversity and socio-economic heterogeneity (Giller et al., 2011). Effective agricultural research for development requires on-farm testing across the full range of diversity so that recommendations can be tailored appropriately (Vanlauwe et al., 2016).

Given that diversity, one of the key challenges is assessing the adoption potential of new technologies. Studies of technology adoption have accordingly begun to focus on farmer preferences and decision-making processes, using a variety of methods, including: social network analysis (Conley and Udry, 2001), agent-based modeling (Berger, 2001), cost-benefit analysis (Egbe and Idoko, 2012), conjoint analysis (Baidu-Forson et al., 1997), and choice experiments (Asrat et al., 2010), among more traditional logit and probit regression models (Moser and Barrett, 2006). These studies have shed light on barriers to technology adoption among smallholder farmers (including labor constraints, upfront costs, mismatch with farmer needs, and the need for training).

However, new insights can be provided by approaches that model adoption as a dynamic process occurring over time. Because technology adoption is temporal, understanding the drivers of technology adoption and dis-adoption is a problem that system dynamic models with their stock-and-flow structure are well suited to answer. System dynamics modeling has been used to model adoption trajectories in a variety of systems (Fisher et al., 2000). However, it has only recently been used to investigate low adoption of smallholder agricultural innovation in Africa (Tambang, 2010), such as conservation agriculture in Zambia (Amelia et al., 2014). The core of these models draw on innovation diffusion theory as described in Rogers (2003), which typically is modeled following the Bass diffusion model (Bass et al., 2000) as a positive feedback loop driven by word of mouth, and a negative feedback loop driven by market saturation.

Beyond information flow, learning and trust are dynamic processes critical to the diffusion of agricultural technologies. Farmers must trust that a technology is effective in order to be interested in it; the more successful examples of the new technology's application that they see, the more likely they are to adopt it. Conversely, if farmers do not see many examples of successful adoption, they are likely to be wary of a technology. This emphasis on trust as a driver of technology adoption in a smallholder farmer context is consistent with empirical data from other fields (Bahmanziari et al., 2003).

Adoption is a decision to choose one option over the alternatives, so modeling adoption dynamically must consider the relative attractiveness of each option. Consumer theory from economics provides the theoretical basis for modeling discrete choices based on consumers' cumulative utility of the set of attributes embodied in a particular choice (Lancaster, 1966). Neoclassical economics assumes that farmers will maximize their utility by choosing the alternative with the highest total utility. Benefits that come in future years are discounted to the present time to account for time preferences. Similarly, benefits with some level of uncertainty must be conditioned by their probability distribution and the level of risk aversion. The discrete choice model allows for estimating the probability of making a choice based on the total utilities of all comparisons (Gensch and Recker, 1979).

Combining system dynamics modeling of diffusion with a discrete choice model allows for decomposing and combining preferences for different attributes of a technology with social processes associated with technology diffusion. All prior studies that have attempted to do this have used conjoint analysis to parameterize preferences (Kopainsky et al., 2012; Santa Eulalia et al., 2011; Schmidt and Gary, 2002). Conjoint analysis, a common tool for marketing, has its foundation in conjoint measurement, which is purely mathematical and has little to do with human preferences (Louviere et al., 2010). In contrast, choice experiments are based on behavioral theory of choice and are better suited to eliciting stated preferences (Hanley et al., 2001).

In this paper, we present a system dynamics model, parameterized with data from choice experiments with Malawian farmers, to depict the *ex-ante* potential adoption dynamics of perennial pigeonpea in smallholder farming systems in Malawi. Perennial pigeonpea represents a new technology, as most Malawian farmers who grow pigeonpea cultivate it as an annual intercrop with maize. However, perennial cultivation is not unheard of, as in some instances, ratooning of pigeonpea is conducted in Malawi, whereby the stem is pruned close to the ground after harvest in anticipation of a second year of growth (Rogé et al., 2016). Perennial management has the potential to improve landscape-scale soil fertility and possibly rehabilitate marginal lands if widely adopted (Glover et al., 2010). Data from choice experiments allowed us to assess the tradeoffs potential adopters would be willing to make in order to begin managing pigeonpea as a perennial.

The research questions we investigated in this study were as follows: *What are the potential dynamics of perennial pigeonpea adoption over time in Malawi, and what characteristics of the perennial technology would likely drive adoption trajectories?*

## 2 Perennial pigeonpea for marginal land in Malawi

Perennial pigeonpea has the potential to address multiple issues by increasing soil nitrogen, reducing soil erosion, reducing labor requirements and providing families with valuable fuelwood through its woody stems.

Pigeonpea (*Cajanus cajan*) is a nitrogen-fixing semi-perennial woody shrub that produces an edible seed and is currently managed as an annual intercrop with maize in southern regions of Malawi for income, nutrition, fuel and soil enhancement (Orr and Mwale, 2001; Orr et al., 2015). It has been promoted in southern Africa for soil fertility enhancement either as an annual intercrop or as a pure stand (Akinnifesi et al., 2009). The nitrogen fixing benefits of pigeonpea are needed to improve maize production in Malawi where a majority of farmers can not afford synthetic fertilizers (Dorward and Chirwa, 2011).

Some farmers in southern Malawi have identified the potential for pigeonpea to be managed as a perennial intercrop through ratooning, meaning that it would be left in the ground for several years, rather than being replanted annually (Rogé et al., 2016). This could confer enhanced soil fertility and labor-saving benefits, but would require farmers to manage pigeonpea differently than they have historically. Rusinamhodzi et al. (2017) found that ratooning pigeonpea intercropped with maize was economically valuable for saving seeds while maintaining or enhancing maize yields.

However, integrating perennial grains in smallholder farming systems are not without potential challenges and tradeoffs. Some of these challenges include weeds, livestock, as well as pests and disease problems. However, with pigeonpea the establishment and weeding would be the same, whether it is managed as an annual or ratooned as a perennial. Perennials may also need to be protected from free-range livestock during the non-growing season, and this is a major concern with pigeonpea in much of southern Africa where dry season crop lands are open-access for community grazing.

Due to their extended growing seasons, perennial grains can provide habitat for both soil and residue-borne pathogens, as well as pests and diseases (Cox et al., 2005; Kantar et al., 2016; Pimentel et al., 2012). However, there is no evidence of disease problems from ratooning in Malawi or Tanzania. Pigeonpea insect pests significantly affect yields when they eat the flowers. These pests typically peak towards the end of the rainy season, which means short duration varieties require greater pest control (Jones et al., 2002). Perennial management of pigeonpea may in some cases reduce pest problems by avoiding pest pressure. Ratooned systems often utilize long duration varieties (180-day maturity, that are harvested several months after maize, towards the end of the dry season) where peak flowering is later in the season.

## 3 Methods

This research aims to assess the adoption trajectory of a technology that is not yet available. Given that empirical data on technology uptake is impossible, we use simulations to understand adoption dynamics based on what is known about the system. By creating a system dynamics model based on the characteristics of perennial pigeonpea, the preferences of Malawian farmers and the social learning of smallholder farming systems, we are able to answer our research questions.

In this section, we first describe the theoretical framework for the model and the equations for its structure. This is followed by how the model was parameterized. We then describe the case study area and the model scenarios simulated.

### 3.1 Theoretical framework and mathematical structure of the model

**Fig. 1:**
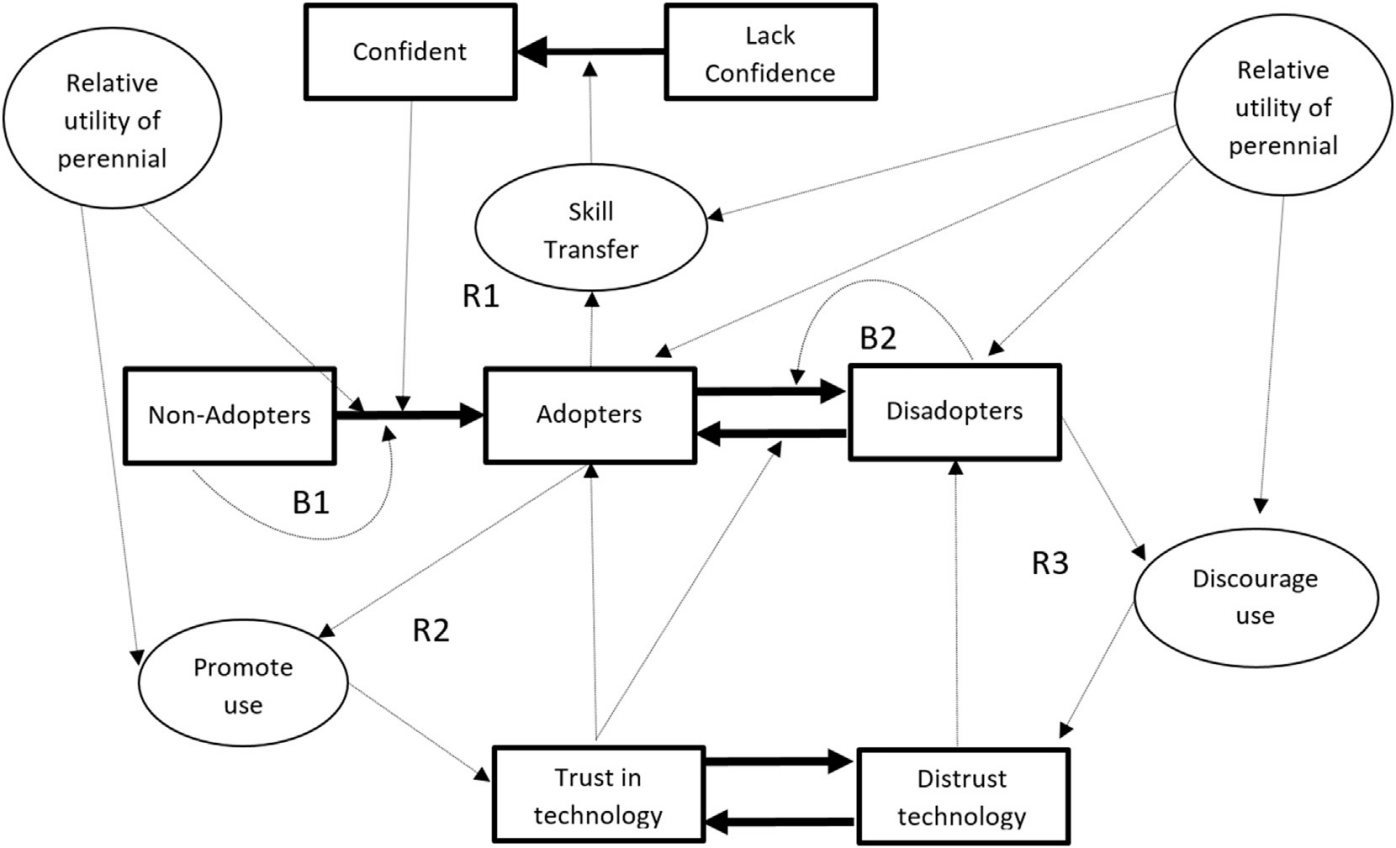
Core structure and causal loop diagram of the system dynamics adoption model. Note: The rectangles are stocks and the arrows with solid lines are flows. The circles and the arrows with fine dashed lines are factors affecting the flows. Reinforcing and balancing feedback loops are labeled with R and B respectively. Notice that “Relative utility of perennial” is included twice to avoid arrows crossing each other.

To answer the research questions described above, we developed a system dynamics model that combines elements of trust in technologies and information sharing with traditional economic elements of utility maximization among discrete choices to simulate the potential demand for perennial pigeonpea over time (Fig. 1). Sociological research on the diffusion of innovations stresses the importance of communication among farmers to transfer skills as well as to share their level of trust in the performance of a technology, which influences the social pressure to conform (Rogers, 2003). This theoretical perspective provides the basis for reinforcing feedback loops for both adoption and disadoption (R1, R2 and R3 in Fig. 1). Many of these dynamics can easily be added to the well-known Bass diffusion model (Bass et al., 2000). A significant improvement on the Bass diffusion model is the inclusion of disadoption and readoption (Ulli-Beer et al., 2010). This allows for a balancing effect on adoption (or re-adoption) as the level of non-adopters (or disadopters) approaches zero (B1 and B2). The combination of the reinforcing and balancing feedback loops leads to the classic s-shaped diffusion curve and is based on social learning theory (Young, 2009).

#### 3.1.1 Utility of annual and perennial systems

The focus of the perennial pigeonpea adoption model is farmers in Malawi who already grow pigeonpea, and their decision whether or not to adopt perennial management of long duration varieties. Here we define adoption as use of perennial pigeonpea on any amount of land. In our model, perennial pigeonpea is a three-year system with two years of ratooning pigeonpea in the dry season (Table 1).

**Table 1:** Annual and perennial production systems by year.

	Annual system	Perennial system
Year 1 – growing season	Maize – pigeonpea intercrop	Maize – pigeonpea intercrop
Year 1 – dry season	Destructive harvest	Harvest and ratoon
Year 2 – growing season	Maize – pigeonpea intercrop	Maize – 2nd year pigeonpea intercrop
Year 2 – dry season	Destructive harvest	Harvest and ratoon
Year 3 – growing season	Maize – pigeonpea intercrop	Maize – 2nd year pigeonpea intercrop
Year 3 – dry season	Destructive harvest	Destructive harvest

The total utility from the perennial pigeonpea system U_p_ is the sum of the utilities from all attributes of the system (maize yield, pigeonpea yield, stem biomass, additional soil fertility and labor savings). Likewise, for the annual pigeonpea system the total utility U_a_ is the sum of the utilities of all attributes of that system (maize yield, pigeonpea yield and stem biomass). We estimated the utilities using data from choice experiments and we estimated the yield and biomass production in two ways: with average values and with stochastic values utilizing APSIM modeled production data from 1980 to 2006 (See Section 3.2 below for details).

One major risk for perennial pigeonpea is that wildlife or free-range livestock may consume it over the dry season before it is harvested. In contrast, farmers harvest short duration pigeonpea varieties closer to when other crops are harvested, thus reducing this risk. We assumed that farmers would only plant perennial pigeonpea in a location where livestock and wildlife would not consume it.

#### 3.1.2 Adoption, dis-adoption and re-adoption

The probability of adoption of the perennial system, Pa, was calculated based on utilities for all attributes of each system (perennial and annual) using the following equation from discrete choice modeling (Gensch and Recker, 1979):

(1) $$\mathrm{Pa}=e^{\mathrm{Up}}/\left(e^{\mathrm{Up}}+e^{\mathrm{Ua}}\right)$$

Where U_p_ and U_a_ are the combined utility from all attributes of the perennial and annual pigeonpea systems respectively (as described above). This probabilistic estimate of adoption is especially suitable for this context because it implicitly models the heterogeneity of farmer-specific costs and benefits. The relative utility of U_p_ and U_a_ are the means for a distribution of costs and benefits of farmers in the simulation. There is a strong basis for this heterogeneity in smallholder farming systems (Giller et al., 2011; Vanlauwe et al., 2016).

The adoption decision in our model is a function of the probability of adoption conditioned by the proportion of confident people who also trust the technology. We also included a constraint for the availability of local seed. Seed limitations were a constraint to more widespread use of improved pigeonpea varieties in Tanzania (Shiferaw et al., 2008). As more farmers adopt perennial management, seed for varieties that work well with perennial management will become more available.

The equation regulating adoption is written:

$$R=N*\left(\left(C*T\right)*P\left(a\right)\right)R=N*\left(\left(C/X\right)*\left(T/X\right)\right)*\mathrm{Pa}\;|\;S>R$$

(2) R=S∣S<R

where R is the rate of adoption (number of farmers/year), N = number of non-adopters (who are potential adopters), C is the proportion of farmers who are confident in their ability to use the technology appropriately (that is, in their ability to manage pigeonpea as a perennial), T is the proportion of farmers who trust the technology, X is the total population, Pa is the probability of adoption, and S is the maximum number of farmers who can adopt based on the amount of seed available from the previous year's production. R is then a flow from the stock N into the stock A for adoption.

Both C and T are stocks whose levels depend on the functions affecting their flows, as represented in Fig. 1 and described below.

##### 3.1.2.1 Modeling disadoption and re-adoption

Disadoption of the technology in our model is the sum of two related but independent effects, which we call direct disadoption (caused directly by disappointment with the performance of the technology) and indirect disadoption (caused by persuasion from disadopters that the technology is not trustworthy or by the desire to conform to the majority).

Direct disadoption is strongest when the relative utility of the perennial is less than that of the annual. In that case, the formula for the direct disadoption rate is one minus the three-year average of the probability of adoption. Using the three-year average is a way to represent farmers' sensitivity to yearly performance but also their familiarity with annual variability. This is the top line of Eq. (3).

Even when the perennial system outperforms the annual system, direct disadoption can occur when farmers are disappointed with the performance of the perennial system relative to their expectations, such as when the relative utility decreases from the previous year. This disappointment effect takes effect when this year's performance is 90% or less of last year's performance relative to the annual. We modeled it as a rate of disadoption and it increases linearly from zero to 10% as the performance relative to last year decreases from 90% to zero. This is the term labeled “Y” in the middle line of Eq. (3). The last line of Eq. (3) is when there is no direct disadoption.

Indirect disadoption happens when social pressures, apart from the technology's performance, influence adopters, such as through influential peers who disadopted or through the more general desire to conform to the majority. Henrich (2001) demonstrated mathematically the importance of these cultural aspects for generating the well-established S-shaped diffusion curve. In our model this is operationalized as first a loss of trust in the technology and then as a desire to conform to the majority, regardless of the relative utility of the technology. First, when disadopters communicate to their peers about their disappointing experience with the technology, they are able to persuade some to lose trust in the technology. The disadopters' level of communication, their persuasiveness, and the social pressure to conform are difficult to measure empirically, and so the values we use in our model are the focus of the sensitivity analysis. We then take the proportion of the population that does not trust in the technology as the indirect disadoption rate, adjusted by a variable representing the pressure to conform, which we also explore in our sensitivity analysis.

Putting it all together the disadoption rate is:

D = A^∗^(1 – ((Pa + Pa_t−1_ + Pa_t−2_)/3)) + A^∗^(L/20) ∣ U_p_ < U_a_

(3) $$D=Y+A^{*}\left(L/I\right)|U_{p}>U_{a}\;\text{and}\;U_{p}/U_{a}<{\left(U_{p}/U_{a}\right)}_{t-1}$$

$$D=A^{*}\left(L/I\right)|U_{p}>U_{a}\;\text{and}\;U_{p}/U_{a}>{\left(U_{p}/U_{a}\right)}_{t-1}$$

Where U_p_ and U_a_ are the combined utility from all attributes of the perennial and annual pigeonpea systems, respectively, Pa… Pa_t-2_ is the probability of perennial adoption at a given time step, and A is the stock of adopters in the previous time step, L is the proportion of the population who lack trust (from the stock in Distrust), I is a variable representing the social pressure to conform, and Y is the disappointment effect described above. *Re*-adoption of the technology is a function of those who disadopted who regained trust in the technology through information from successful adopters. The formula is simply the proportion of the population who gained trust multiplied by the number of disadopters.

#### 3.1.3 Confidence

We modeled the development of skills and confidence as a function of mentoring provided by adopters to communicate the complex skills for perennial management. We assumed a base skill transfer rate of 10 adopters training one new farmer each year and a maximum of one adopter training two farmers each year, depending on the relative utility of the perennial to the annual system.

Next, given the importance of risk aversion for smallholder technology adoption (Feder, 1980; Ghadim et al., 2005) we assumed that a maximum of 80% of those who gained the skills to manage the perennials in a given year would have the self-efficacy to change their practice (i.e. they would be confident enough to try it). This willingness to risk trying a new technology is also modeled to be a function of the relative utility of the annual and perennial systems, specifically that those confident to use their skills would increase linearly from 40% when the two systems are equal to 80% when the perennial system has double or more the utility of the annual system. We assumed that the obtaining skills and confidence was permanent.

We assumed that some outside force (such as development project) would initiate promotion and be able to provide 10 farmers per year with sufficient confidence and trust in the technology that they would adopt. We assumed this promotion would happen only when 10% or less of the population was using the perennial system. This exogenous promotion was essential for catalyzing the initial adoption trajectories in our model.

#### 3.1.4 Trust

We modeled trust as a reversible process that is a direct function of farmers' experiences communicated to their peers. We adapted the structure for these dynamics from models that focused on adoption of improved seed in Africa based on trust in brand names (Derwisch et al., 2011; Kopainsky et al., 2012; Kopainsky and Derwisch, 2009). We assumed that each adopter would talk to ten of their peers per year about their experiences (Kopainsky and Derwisch, 2009) and that their persuasiveness would be equal to the three-year average probability of adoption. We also assumed that each non-adopter would contribute to the development of trust by discussing the new technology with ten peers though with very low persuasiveness compared to that of adopters (1%). Considering disappointment may lead to strong emotions, we assumed that each disadopter would communicate their experience to twenty peers per year, with their persuasiveness being equal to one minus the three-year average probability of adoption. Given the highly uncertain nature of the risk aversion, trust, and communication variables, and the lack of empirical data from the specific study population, we conducted an extensive sensitivity analysis on these parameters to determine the significance of their contribution to model outcomes (see Section 4.2 and Fig. 6, Fig. 7).

### 3.2 Model parameterization

All simulations consist of 1000 households already growing annual pigeonpea with annual decisions about the use of perennial management over 25 years, which provides ample time to observe patterns of adoption and interannual yield variability. The values for key model parameters and their sources are presented in Appendix 1.

#### 3.2.1 Agronomic parameters

The complex impacts of perennial management of pigeonpea on production of both pigeonpea and maize over time are not well researched. Pigeonpea is more drought tolerant than maize and perennial pigeonpea has a deeper root system in its second and third year, which makes it even more drought tolerant.

To parameterize our model we draw on recent research using APSIM modeled maize-pigeonpea intercrop production in Malawi (Smith et al., 2016) and ratooned maize-pigeonpea intercrop trials in Tanzania (Rusinamhodzi et al., 2017). We combine the APSIM modeling results from Smith et al. (2016) with the ratoon effect from Rusinamhodzi et al. (2017) to estimate maize and pigeonpea yield across seasons for the annual and perennial management systems. We also use data from Gwenambira (2015) to estimate woody pigeonpea biomass.

Smith et al. (2016) used APSIM to model several maize-legume systems at Africa RISING sites in Malawi, including intercropped maize and pigeonpea (managed as an annual). That model used historic rainfall data from 1980 to 2006. Using the APSIM modeling output for the low potential maize site in Malawi (Golomoti) we developed formulas for the upper and lower yields for maize and pigeonpea for any given season quality.

We created a season quality index using the sole maize system as a proxy by calculating each season's sole maize yield as a proportion of the average sole maize yield from the 1980 to 2006 seasons. The result is a variable between 0.2 and 1.6 that has a beta distribution and our stochastic model randomly selects a seasonal index from that distribution for each year. Each year our model then selects a random value from a uniform distribution between the upper and lower yield limits for each crop based on the APSIM model. The baseline model has the season index fixed at 1 (average season).

**Fig. 2:**
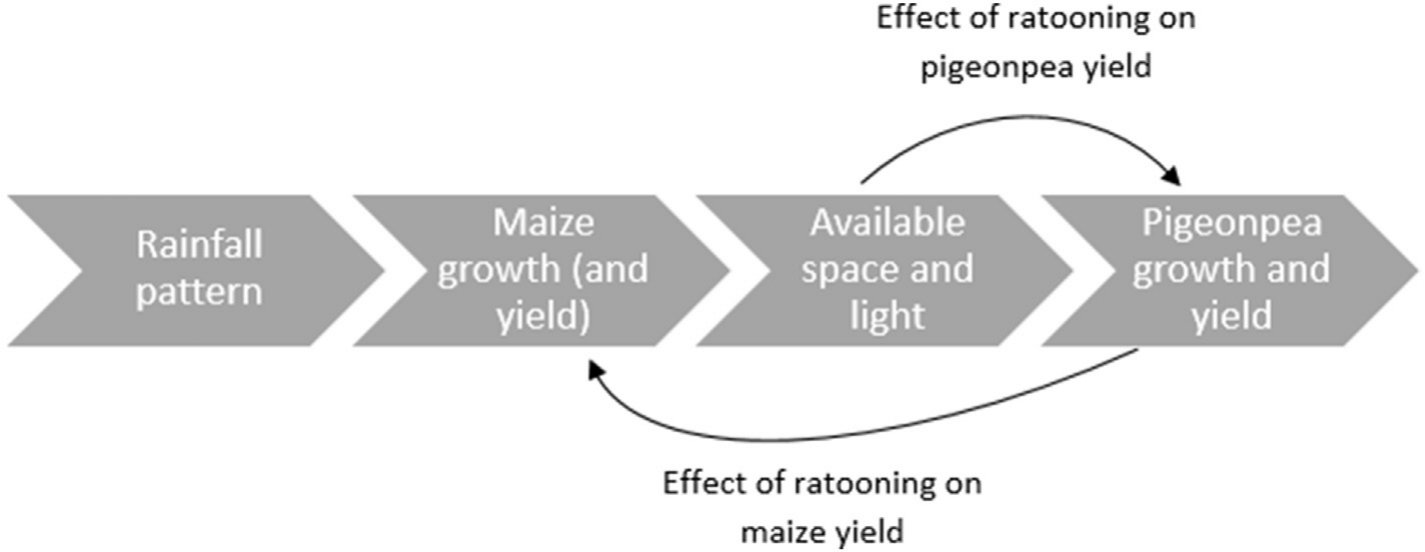
Dynamic interdependencies between maize and pigeonpea yields driven by seasonal rainfall.

The model simulates maize and pigeonpea yields according to a logical flow of interdependent relationships between the two crops, driven by seasonal rainfall (Fig. 2). We assume that rainfall patterns are driving maize production, which in turn dictates the available water and light for pigeonpea.

Rusinamhodzi et al. (2017) provide data on yields from ratooned pigeonpea intercropped with maize in Tanzania. That study contains observations from two years of ratooned pigeonpea and shows that on the dry year, the ratooned system had higher pigeonpea and lower maize yields than the annual system. However, on the wetter year, the ratooned system had lower pigeonpea and higher maize yields than the annual system.

**Fig. 3:**
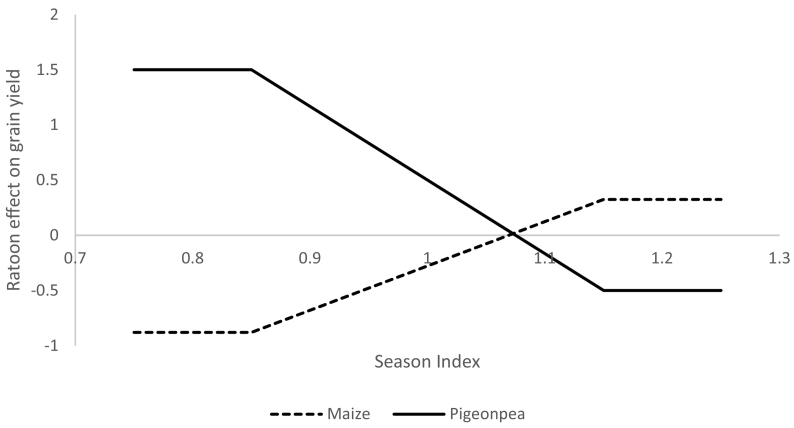
Effect of ratooning on maize and pigeonpea yield across season quality.

We used that data to create season-dependent non-linear effects of ratooning on pigeonpea and maize yields. Using the data between ratooning and season from Rusinamhodzi et al. (2017) we calculated the ratoon effect on maize and pigeonpea for a very dry season and for a very wet season and then estimated that a normal season would lie between these values (Fig. 3). In our model, we calculated the yields for the perennial system by multiplying the APSIM modeled yield for each crop by the crop-specific ratoon effect.

#### 3.2.2 Farmer preference parameters

Farmers' preferences for the attributes that change from annual to perennial management of pigeonpea were modeled using the results of a choice experiment (Waldman et al., 2017), an increasingly common method in development economics for obtaining stated preferences among hypothetical scenarios (e.g. Pienaar et al., 2014; Roessler et al., 2008; Schipmann and Qaim, 2011; Ward et al., 2014). The choice experiment was designed based on a series of focus groups with farmers and interviews with experts in Malawi's Central and Southern regions to identify the most important attributes and tradeoffs involved in annual versus perennial pigeonpea production. Waldman et al. (2017) identified the tradeoff between maize and pigeonpea yield as a key factor in farmers' decision-making process as well as the length of time the crop was in the field, the degree to which it improves soil quality, and how much biomass is produced. The choice experiments were based on an orthogonal experimental design of these attributes using illustrated booklets where farmers evaluated a series of five choice sets. Coefficients were estimated using a random parameters logit model and these are marginal utilities we used to parameterize our system dynamics model. Full details on the choice experiments are available in Waldman et al. (2017).

We used the results of the choice experiments to derive utilities for each attribute of the perennial system and we imputed utility values for the annual system by multiplying the marginal utility per kg from the choice experiments by the total production of grain for each crop (see Appendix 2 for details).

The choice experiment necessarily simplified differences in biomass into “high” and “low”. To convert this into a continuous variable, we assumed that farmers' perception of the difference between these categories was accurate and then calculated a marginal utility per kg of additional biomass. This further assumes that farmers' utility for an additional kg of biomass is constant across the full range of observed biomass. While this assumption may not be appropriate for levels of biomass beyond the consumption needs of the household, it is an appropriate simplification in this case given the relatively small areas dedicated to pigeonpea and the high demand for biomass at the household level.

### 3.3 Model validation

There is no empirical data on uptake of this technology because it is not being practiced at any measurable scale in Malawi. To ensure that our model is providing reasonable results we engaged in expert consultation with agronomists, economists and development practitioners who are familiar with pigeonpea in Southern Africa. We also presented an early version of the model to international scientists working on perennial grains at a meeting in Bamako, Mali in 2015. Furthermore, we subjected our model to a series of extreme behavior tests to ensure reasonable expectations when either technology is clearly superior. Finally, the adoption of perennial pigeonpea over 25 years is reasonable given how annual pigeonpea diffused through southern Malawi from very low levels in 1990 to fairly high levels at present in response to market opportunities. Introducing new management of a known crop has fewer barriers than introducing a brand new crop. For this reason, we limit our model's application to farmers already growing annual pigeonpea. Many other factors would need to be considered for adoption in areas not already growing annual pigeonpea.

### 3.4 Model simulation runs

In our baseline model runs we simulated adoption with average, constant yields for maize and pigeonpea for the duration of the simulation. This simple version of the model allows for the analysis of how various scenarios are likely to affect adoption trajectories. We present results for various scenarios by systematically changing maize yield potential, pigeonpea yields and livestock pressure.

Next, we simulated adoption of a more realistic representation of the agricultural system by including variability in yields and season quality with their corresponding impact on the relative performance of the perennial system. A single run of this simulation provides little information for comparisons. For this reason, we ran 1000 simulations for each comparison and we present the mean, min and max values from each year.

### 3.5 Methods used for sensitivity analysis

We used a systematic approach to sensitivity analysis by gradually varying key factors and assessing their influence on the adoption trajectories. By changing the biophysical variables, such as the average crop yields in the baseline model runs, we can explore how adoption rates would change with the agro-ecological potential of an area. We also present results when dry season livestock destroy the pigeonpea above ground biomass, thereby eliminating the pigeonpea harvest and the soil fertility benefits from decomposed leaves.

We also carried out sensitivity analysis on the social variables including the importance of conformity as well as the persuasiveness and communication rate of disadopters, adopters and re-adopters.

## 4 Results and discussion

**Fig. 4:**
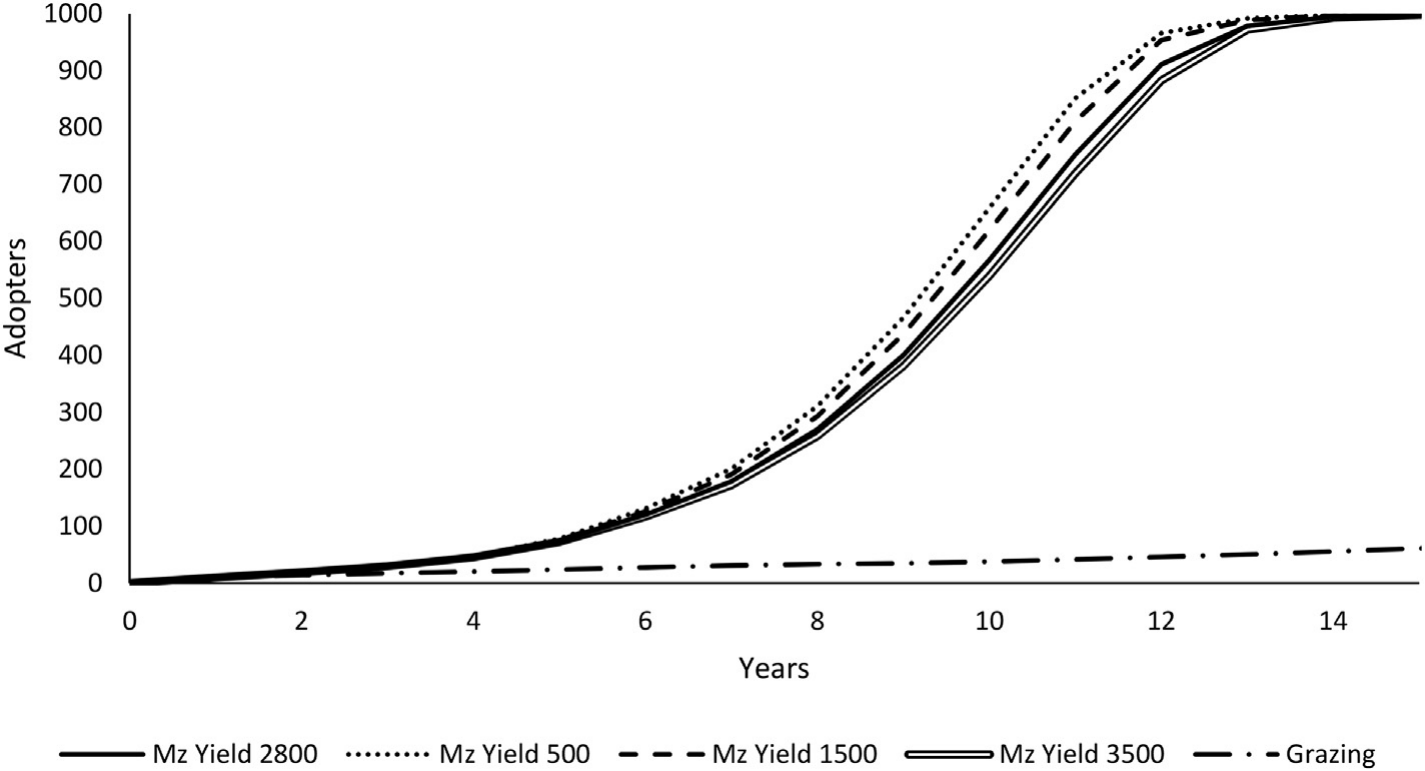
Adoption pattern for the full range of average maize yields using the baseline model parameters (constant yields and constant “normal” seasons).

Under the baseline model run with average yields (2800 kg ha^−1^ maize and 289 kg ha^−1^ pigeonpea), farmers are estimated to have a probability of adoption of the perennial system of 77%. However, the newness of the technology causes adoption to occur gradually as people gain trust and skills in the perennial management of the crop. This results in a diffusion curve that reaches 77% of pigeonpea farmers adopting by year 12 and approaching saturation (100% of farmers) by year 14 (Fig. 4 – solid line).

### 4.1 Conditions for adoption of perennial pigeonpea

Simulations with lower than average maize yields have slightly faster rates of adoption (Fig. 4) because in these conditions the relative benefit from the perennial system is greatest. The choice experiments indicate that farmers generally value maize more than pigeonpea grain. When maize yields are higher the cost of reduced maize yields due to competition from the perennial are smaller, making the perennial system more attractive. When we include animal grazing (which eliminates the pigeonpea harvest, the soil fertility and biomass benefits) adoption is minimal throughout the simulation (Fig. 4).

When we doubled the utility from pigeonpea grain (the equivalent of doubling the price or yield), adoption rates increased only slightly (not shown). This reflects farmers' higher utility for maize relative to pigeonpea grain and suggests that the key value of the pigeonpea to farmers is the soil fertility enhancement it provides for maize.

**Fig. 5:**
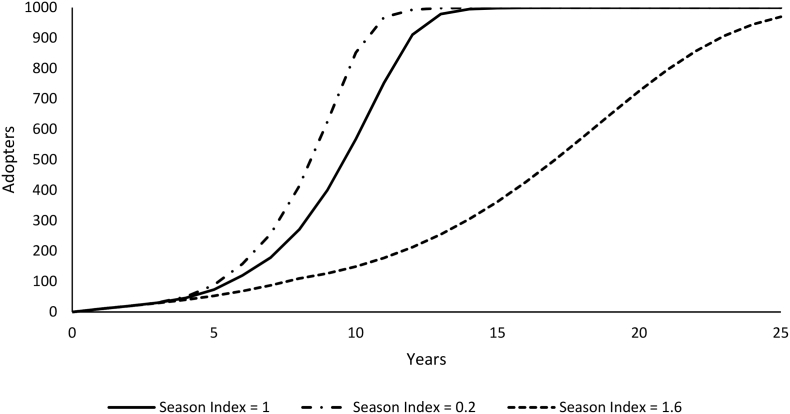
Adoption pattern across the range of season quality for maize (fixed for the entire simulation) using constant average yields.

Scenarios with constant seasons that are worse than average for sole maize, which tend to be seasons with dry spells, have faster adoption rates, because in these conditions the maize yields are lower and so the cost of competition from the perennial system is lower (Fig. 5). Scenarios with better than average seasons for sole maize, which tend to be wetter seasons, have significantly suppressed adoption because in that context the additional maize production benefits are offset by reduced benefits from pigeonpea grain and biomass, though overall the perennial system still has higher utility.

This shows that the ratoon effect by season is a major driver of the adoption pattern. This is interesting because the perennial system has higher utility than the annual system across all values of the season index. This highlights how adoption may be very slow for technologies that are only slightly better than the status quo due to the informational uncertainty.

**Fig. 6:**
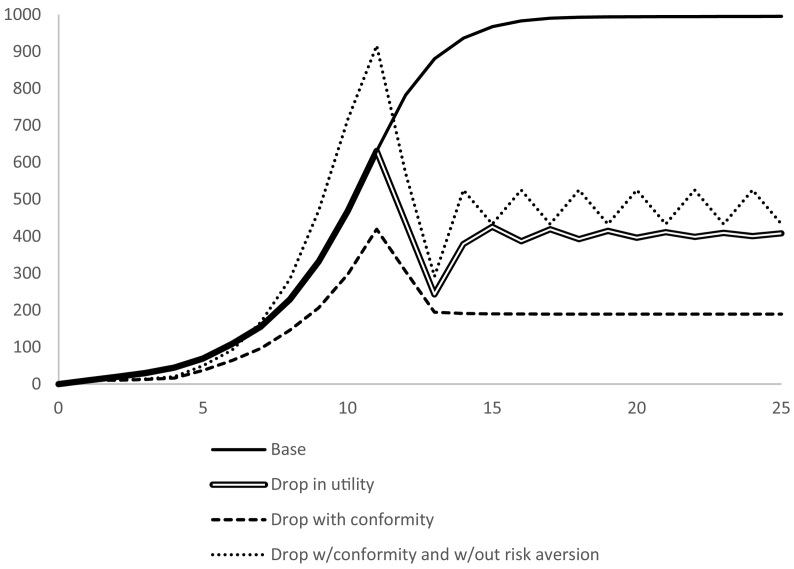
Adoption trajectories with a sudden drop in utility at year 10 under various scenarios of conformity and risk aversion.

**Fig. 7:**
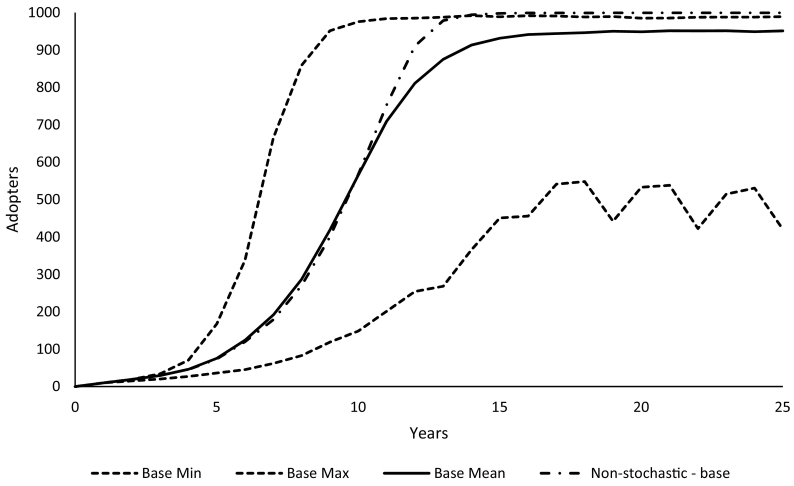
Adoption trajectories with and without stochastic yield and seasonal variability affecting production.

A sensitivity analysis on the social variables in the model revealed that their effects on the adoption trajectory were largely limited to scenarios where adoption was slow or variable. Most of these scenarios included stochastic yields and seasons, but sensitivity without stochasticity was also explored (see Fig. 6). The disadoption communication rate and persuasiveness had the largest influence on the average adoption level (after 1000 iterations), while the adoption and non-adoption communication rates had small effects. The factors for social conformity, disadopters communication rate and disadopters' persuasiveness were synergistic in causing a non-linear tipping point. If these three values were all high enough (double the default value) then adoption was significantly suppressed but a slight reduction in any one of them caused much higher adoption. These higher values are used in the “conformity scenario” for Fig. 7.

### 4.2 Effects of variability in production and climate on adoption trajectories

Before exploring stochasticity in production and utility, we present how the model responds to a single permanent shift in the utility of the perennial system whereby on average farmers' utility for it is much less than the annual system (relative utility shifts from 1.2 to −1.0). Such a shift would be similar to a real-world scenario that makes the production of the perennial much more costly, such as a new alternative dry season land-use opportunity. The drop in utility causes an immediate drop in adoption, followed by a small recovery as the peer pressure from disadopters wears off and the conformity of those who continue using the perennial system encourages others to continue trying the perennial system (double line in Fig. 6). Higher social pressure to conform eliminates the recovery from the initial drop. Interestingly, a reduction in risk aversion makes the recovery stronger. When high social pressure to conform is combined with reduced risk aversion, there is an oscillation pattern resulting from the tension between two reinforcing feedback loops – the adopters communicating their success and the disadopters communicating their discouragement (R2 and R3 in Fig. 1). In all of these scenarios the response to the sudden drop reaches a new stability after 3 or 4 years.

Having stochastic yields and stochastic seasons in the model has a surprisingly large effect on the range of potential adoption trajectories, especially because the average utility for the perennial system never goes below zero. The minimum values from 1000 iterations were much lower and are the collection of the low points from oscillations in disadoption and readoption. The average from 1000 iterations was similar to the non-stochastic baseline scenario, though plateauing at a lower level (Fig. 7). Perennial pigeonpea outperforms annual pigeonpea under the full range of climatic conditions but the amount by which the perennial system outperforms annual pigeonpea is marginal in poor seasons, and this marginal improvement is not enough to overcome the inherent risk aversion farmers demonstrate around a new and transformative technology. Furthermore, disadoption after a poor rainfall year results in a loss of trust in the perennial system, which is only regained gradually with better seasons.

**Fig. 8:**
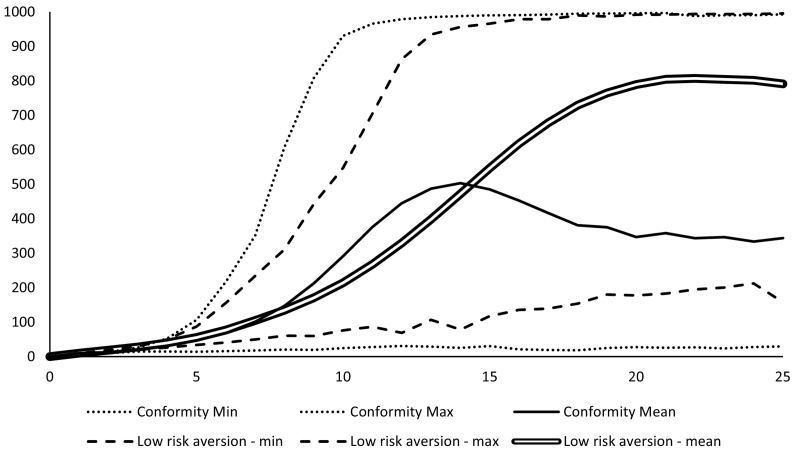
Adoption trajectories with increased levels of conformity and lower levels of risk aversion.

Combining that stochastic baseline scenario with a higher degree of conformity and a greater persuasiveness of disadopters led to average adoption levels of less than 40% (Fig. 8 – solid line). The conformity scenario affects the latter half of the adoption trajectory to a greater extent because of the stronger influence of disadoption on levels of trust as adoption levels increase. The disadoption is initially caused by decreased relative utility of the perennial system in certain seasons but then triggers further disadoption and slower adoption because of a loss of trust in the perennial system.

Poor seasons therefore suppress the adoption trajectory in contexts with high cultural conformity. This effect is less pronounced when yields and seasons are held constant at the average. When farmers are less risk averse but conformity is still high the average adoption trajectory is significantly increased (Fig. 8 – double line) but still below the base scenario with risk aversion but low conformity. This suggests that reducing the risk of trying new technologies may be one strategy for reducing the effect of social pressure to conform. Risk aversion in our model affects adoption as the proportion of those who gain skills that are willing to implement them. Thus, a similar effect could be achieved by strengthening the quality of training and linking it better to implementation.

A key finding of this study is that the inherent stochasticity of agricultural conditions limits agricultural technology adoption rates in cultural contexts of conformity and risk aversion, even when these technologies present clear advantages over existing systems. The stochasticity enters through the probability of adoption, which is a function of the relative utility of perennial pigeonpea compared to annual pigeonpea.

Overall, adoption patterns are driven primarily the season-specific benefits of the perennial system which motivate adoption and stimulate reinforcing feedback loops that build trust and skills for perennial management (R1 and R2 in Fig. 1). In addition, adoption dynamics involve social feedback, so that poor seasons lead some to disadopt and these disadopters communicate their negative experiences, triggering a loss of trust, which can further disadoption (R3 in Fig. 1). This dynamic would apply to any new agricultural technology whose relative advantages over traditional practices is dependent on climatic variability, which is virtually any technology that seeks to improve yields. This finding has clear explanatory value for the common phenomenon of low adoption rates among risk-averse farmers of technologies that have yield-boosting potential (Friedlander et al., 2013; Giller et al., 2009).

## 5 Conclusions and implications of the research

The combination of choice experiments, crop simulation modeling and system dynamics modeling is uniquely powerful to investigate the adoption trajectories of new technologies. This approach allowed us to use modeling to project technology adoption into the future, while grounding our projections in empirical data about farmers' preferences. The use of this method led to insights about one potential cause of low adoption rates of technologies that can clearly improve yields for smallholder farmers—namely, the social feedback effects of dampened adoption rates in years in which these technologies represent only modest gains in productivity, due to climatic stochasticity.

Confronting the complexities of social dynamics affecting adoption is key to supporting sustainable intensification of agriculture. There are three major implications from this study. First, policy makers should be made aware that climatic variability makes it difficult for farmers to evaluate the “average” effect of technologies and therefore the new practices may not be adopted as well as might be expected from initial research trials and surveys of farmer preferences. Second, trust in a technology is a complex social process that cannot be created among project participants simply through demonstrations and training. Peer to peer extension strategies combined with the availability of expert support to address farmers' concerns may facilitate the development of trust in new practices. Finally, promoters of a new technology should be aware of the power of disadopters to dampen adoption trajectories by instigating loss of trust in the technology. Development agencies can mitigate this by taking disadopters' concerns seriously and helping farmers have clear expectations about any technology's sensitivity to factors outside farmers' control, such as climate and markets.

Testing the actual magnitude of the effect of social dynamics on adoption trajectories will require focused research efforts. We encourage a time horizon of at least several years for studies that track agricultural technology adoption, in the context of the climatic conditions experienced by technology adopters. While this modeling study is based in empirical observations of farmers' preferences, the evolution of those preferences over time, and their response to changing socio-economic and ecological conditions, are more speculative. Generally, there is a dearth of longitudinal studies of technology adoption dynamics in sub-Saharan Africa.

## Figures and Tables

**Table:** 

Description of variable	Value	Sources
Agronomic variables		
Mean pigeonpea yield – annual or 1st year perennial	267.7 kg ha-1	Snapp et al., 2002 – mean of 3 sites
Mean second year pigeonpea yield - perennial	436 kg ha-1	Interpolating Rusinamhodzi et al., 2017 for an average season
Seed savings for perennial system	6.6 kg ha-1	Seed weight from (Narayanan et al., 1981), Planting density from Gwenambira, 2015
Pigeonpea grain harvest index	0.2	Rogé et al., 2015 Table 5 p.68
Mean maize yield when intercropped with pigeonpea - annual or 1st year perennial	2845.8 kg ha-1	Smith et al., 2016

Farmer preference variables		
Marginal utility of labor saving with perennial (considering also the risk of crop loss)	0.164	Waldman et al. (2017) Table 3 Preference space – random parameter means
Marginal utility of higher soil fertility in year following perennial pigeonpea	0.768	Waldman et al. (2017)
Marginal utility of higher biomass production from perennial pigeonpea	0.358	Waldman et al. (2017)
Marginal utility of another kg of pigeonpea grain	0.015	Waldman et al. (2017)
Marginal utility of another kg of maize grain	0.023	Waldman et al. (2017)

Trust and communication variables		
Base skill transfer rate – the number of people each adopter could train in a year	0.1 to 2	Kopainsky and Derwisch, 2009
Base proportion of those trained who would be confident enough to try the new practice	0.4	Based on experiences with Malawian farmers and literature on risk aversion (e.g. Grothmann and Patt, 2005)
Social pressure to conform	0 to 0.1	Buffering factor for indirect adoption loosely related to communication rates (1/10 = 0.1) based on social network size.
Disadopter communication rate (the number of people they tell about their experience each year)	20	Based on Kopainsky and Derwisch, 2009
Adopter communication rate (the number of people they tell about their experience each year)	10	Kopainsky and Derwisch, 2009
Non-adopter communication rate (the number of people they tell about the positive things they have heard about the technology)	10	Kopainsky and Derwisch, 2009
Persuasiveness of non-adopters (the proportion of those convinced to trust in the technology)	0.01	Kopainsky and Derwisch, 2009

**Table:** 

Attribute	Levels	Description
Perennial	Yes, No	Whether the pigeonpea was managed as a perennial crop
Soil fertility	Low, high	The level of soil fertility in the following year
Biomass	Low, high	The level of biomass produced over the planting period
Pigeonpea yield	50, 100, 150, & 200	Yield of pigeonpea in kg per 0.5 acres
Maize yield	150, 200, 250, & 300	Yield of maize in kg per 0.5 acres
